# Comparing the Efficiency of a Newly Designed Spring With the T Loop for Buccal Canine Disimpaction: A Finite Element Study

**DOI:** 10.7759/cureus.45264

**Published:** 2023-09-14

**Authors:** Harsha L, Prasanna Aravind TR, Saravanadinesh SP, Shweta Nagesh

**Affiliations:** 1 Orthodontics and Dentofacial Orthopaedics, Saveetha Dental College and Hospital, Saveetha Institute of Medical and Technical Sciences, Saveetha University, Chennai, IND

**Keywords:** finite-element model, maxillary impacted canine, spring, loops, orthodontic tooth movement, rate of tooth movement

## Abstract

Disimpacting a buccally impacted canine precisely using frictionless mechanics is a challenge in orthodontics. Various mechanics can be used for aligning an impacted canine and a spring is one of the most versatile methods of accomplishing it. The present study describes a newly designed spring for canine disimpaction using the finite element method (FEM) model. In the present study, the efficiency of the newly designed spring was compared with the T loop. The FEM model of the maxilla was prepared. Harsha's Canine Disimpaction (HCD) spring and T loop with no pre-activation bends were utilized to disimpact the left maxillary canine using a 0.017 x 0.025 inch titanium molybdenum alloy wire. Efficiency of both springs in all dimensions (sagittal, vertical and transverse) was compared. Stress acting on the bone and teeth and number of activations needed for disimpaction were evaluated when the spring was activated by 3 mm. A movement/displacement of 0.8, 0.4 and 0.1 mm was seen in the sagittal (X), transverse (Y) and vertical (Z) planes, respectively, with the HCD spring, and 0.4, 0.1 and 0.1 mm seen in the X, Y and Z planes, respectively, with the T loop. A total of 12-13 and 80 activations were required as per the FEM simulation for disimpaction using the HCD spring and T loop, respectively. Stress concentration in the disto-cervical region was observed during disimpaction with both HCD spring and T loop. Within the constraints and limitations of the present study, it can be concluded that the HCD spring requires lesser activation and brings about greater control of canine disimpaction in all three dimensions when compared to the T loop.

## Introduction

Maxillary canines are the second most common impacted teeth after third molars with a prevalence rate of 1%-3% [1]. They can be treated orthodontically by disimpaction using various traction techniques or springs that utilize frictionless mechanics to deliver constant extrusive force systems. Multiple springs, utilizing optimal force-moment ratios have been elucidated in the orthodontic literature for the delivery of constant forces [1-4]. However, most of the described springs are predominantly used for palatally impacted canines considering the universally acknowledged difficulties encountered with direct traction techniques. Buccal disimpaction involves a wider variety of treatment protocols with monkey hooks, gold chains, elastomeric chains and ligature ties utilized in direct traction. Recent advancements in implant-aided retraction techniques have reduced the anchorage strain encountered in the dentition and simplified treatment mechanics [5,6].

The greatest problem encountered by a practitioner during buccal canine disimpaction is the challenge in maintaining the periodontal support surrounding the anterior dentition. Indiscriminate force application, or inadequate anchorage preparation can result in arch form distortion, proclination of anterior teeth, along with gingival defects manifesting as pockets and dehiscence. Frictionless mechanics utilized for buccal canine disimpaction require proper posterior support along with constant force systems that provide significant versatility in their designs and fabrication types. A varied number of springs and loops such as T loop, mushroom loop, spec loop, Paul Jessing spring, L-loop and keyhole loop [7-10] have been widely used for individual canine retraction. These loops ideally produce a high moment/force ratio, low force/decay rate and are easy to fabricate. However, the structural limitations of these loops mean that the ideal loop generating the above-mentioned characteristics does not exist. As a fail-safe and convenient loop, the T-spring has been the most commonly used loop for retraction [11-14] and space closure. In the case of canine disimpaction, however, the T loop can cause trauma to the vestibular mucosa due to impingement and soft tissue overgrowth. It is with keeping these limitations in mind, a novel HCD spring, or Harsha's Canine Disimpaction spring, was designed to provide a malleable and fail-safe loop in canine retraction. The aim of this study was to compare the efficiency of the novel HCD spring and the commonly used T loop in terms of number of activations required during treatment and stresses acting on the surrounding bone during buccal disimpaction of canines. The stresses and force vectors utilized in the study were standardized to ensure identical case scenarios.

## Technical report

HCD spring

**Figure 1:**
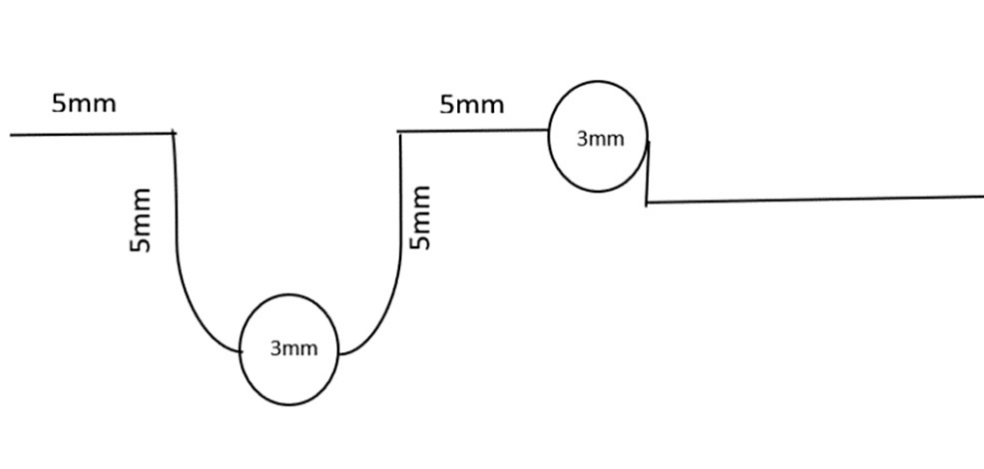
Schematic diagram of the HCD spring HCD spring, Harsha's Canine Disimpaction spring

The HCD spring consists of three horizontal arms, two vertical arms of 5 mm and two helices of 3 mm diameter coiled together. The two helices were made to represent both the sagittal and vertical planes of space by ensuring one horizontal and one vertical arm between them to generate maximum biomechanical efficiency (Figure 1).

**Figure 2:**
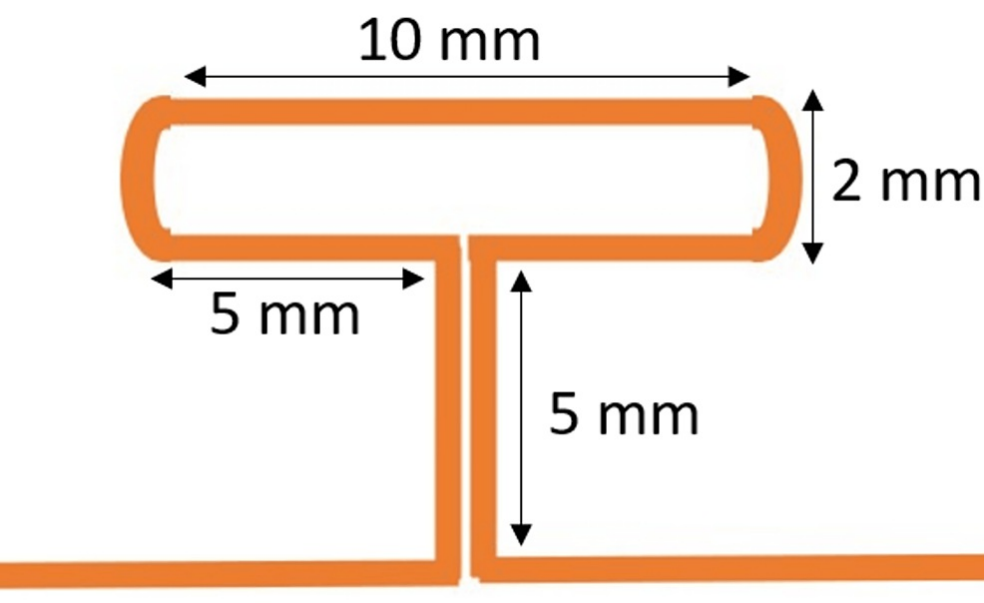
Schematic diagram of the T loop

It was made with 0.017 x 0.025 inch titanium molybdenum alloy (TMA). The anterior portion of the spring was inserted into the maxillary canine bracket slot and the posterior portion was inserted into the auxiliary slot of the molar buccal tube. The T spring was made with 0.017 x 0.025 inch TMA with dimensions and bends given in corroboration with Burstone (Figure 2) [15]. Both the springs were activated by extending the beta end distally by 3 mm and cinched back tightly.

**Figure 3:**
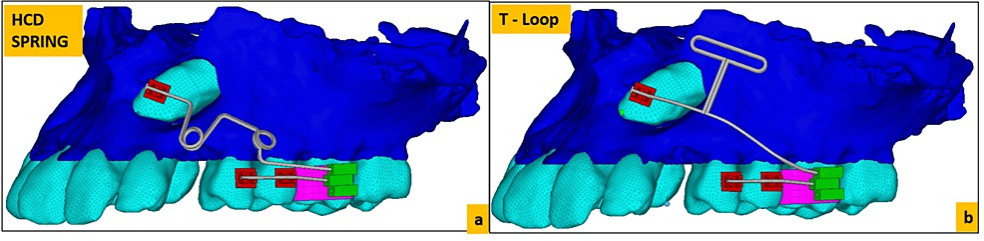
(a) Mesh model of the HCD spring; (b) mesh model of the T loop HCD spring, Harsha's Canine Disimpaction spring

Two different finite element method (FEM) models were prepared for the HCD spring and T loop to apply 60 g of force to a unilateral buccal impacted maxillary canine tooth. The FEM model of the maxillary arch was prepared using a cone beam computed tomography (CBCT) scan of a patient with an impacted canine in the second quadrant. The CBCT scan was taken with Carestream CS 9600 (Carestream Health, Inc, Rochester, NY) by a single operator with standard settings where the patient was standing in an upright position verified by the artificial intelligence (AI)-integrated positioning system. The field of view (FOV) of the retrieved scans was 8x8 with a three-dimensional view of only the maxilla; the parameters at which the scans were taken were 0.7 mm Cu, 120 kV, 4.0 mA, 150 seconds and 75 mGy.cm^2^. Patient consent for the use of the CBCT scan was obtained. The obtained scan in DICOM format was transformed to STL files using MIMICS 8.11 software (Materialise NV, Belgium). Using SolidWorks software (Dassault Systèmes SE, France) with the 3D EXPERIENCE platform, geometric models of the loops were constructed with lines and surfaces using certified workstations. On importing the geometric model into Abaqus software (Dassault Systèmes SE), the 3D finite model was made; HyperMesh software (Altair® HyperMesh®; Altair Engineering, Troy, MI) was used to obtain precision (Figure 3).

In the FEM model, the smallest unit was divided into shapes called "elements", which are expressed as simple geometric models and serve to maintain a constant distance between the nodes to which they are connected. By dividing the models, a finite number of elements are connected to one another at certain points, and these points are called nodes. In the models, displacements are associated with displacements in each element. The final model contained 229,889 nodes and 748,409 elements for the HCD spring, and 232,069 nodes and 749,861 elements for the T loop.

In the FEM model, McLaughlin-Bennett-Trevisi (MBT) brackets with a 0.022 x 0.028 inch stainless steel (SS) slot prescription bracket were placed on the maxillary premolars and molars and the segmented arch was stabilized with a 0.19 x 0.025 inch SS archwire. The buccal surface of impacted canine was exposed on the model and bonded with the same prescription bracket. The model consisted of a retained deciduous canine, which was removed and the obtained space was filled with bone in order to simulate normal anatomic structure. Properties of teeth, periodontal ligament (PDL), bone, wires and brackets in the prepared FEM model are given in Table 1.

**Table 1:** Properties of teeth, periodontal ligament, bone, wires and brackets in the prepared FEM model FEM, finite element method

Properties	Wire and loop	Maxillary bone	Teeth	Periodontal ligament	Bracket
Young’s modulus (MPa)	69,000	2000	20,000	5	200,000
Poisson’s ratio	0.4	0.4	0.4	0.4	0.4

Principal stresses and displacements in three directions of space were determined by FEM analysis. Principal stresses were used for fragile materials (bone, teeth, etc.). Total displacement and displacement values in the X, Y, and Z directions were determined in the measurements of the HCD spring and T loop models. For X direction displacement (sagittal), the plus value indicates distal displacement of canine; for Y direction displacement (transverse), the plus value indicates palatal displacement of the canine and for Z direction displacement (vertical), the plus value indicates the extrusion movement of the canine. The limiting conditions for movement were applied such that they allowed movement of the alpha terminal (the end attached to the canine) in all three planes while the beta node was restrained from any movement. No pre-activation or anti-rotation bends were incorporated in the springs. The magnitude of tooth movement in the sagittal (X), transverse (Y) and vertical (Z) planes, along with the stresses acting on bone and teeth, were calculated for both the loops using Abaqus software. The number of activations needed for complete disimpaction of the canine was also calculated using the FEM model.

Results

**Figure 4:**
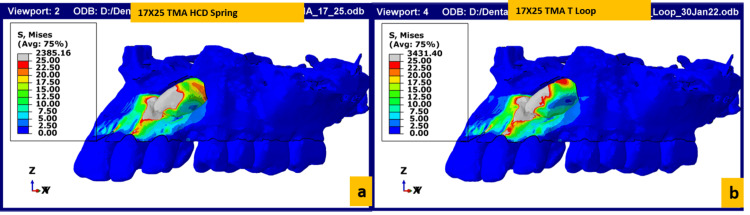
Stress acting on the bone and teeth with (a) HCD spring activation and (b) T spring activation HCD spring, Harsha's Canine Disimpaction spring; TMA, titanium molybdenum alloy

The stresses acting on the bone and teeth, i.e., the von Mises stress for the two springs, are depicted in Figure 4. As per the FEM model simulation, it can be observed that there is increased stress concentration in the disto-cervical region of the tooth at the cementoenamel junction. There is increased stress concentration on the bone present around the mesio-incisal aspect closer to the tip of the canine. The stress distribution in the T loop was greater than that exerted by the HCD spring.

Details on the amount of canine disimpaction in all three planes are given in Table 2. In the present study, the HCD spring brought about twice the amount of tooth movement when compared to the T loop. The HCD spring brought about a movement of 0.8 mm in the X (sagittal) plane, 0.4 mm in the Y (transverse) plane and 0.1 mm in the Z (vertical) plane when fabricated using a 0.017 x 0.025 inch TMA wire and 3 mm activation. When the same degree of activation was carried out with a T loop, 0.4 mm movement of the canine was observed in the X (sagittal) plane, 0.1 mm in the Y (transverse) plane and 0.1 mm in the Z (vertical) plane.

**Table 2:** Amount of canine displacement in all three planes of space TMA, titanium molybdenum alloy; HCD spring, Harsha's Canine Disimpaction spring A 0.017 x 0.025 inch TMA wire was used.

S. no.	Loop type	Displacement		
		X direction (mm)	Y direction (mm)	Z direction (mm)
1	HCD spring	0.802	-0.412	-0.149
2	T loop	0.416	-0.163	-0.117

**Figure 5:**
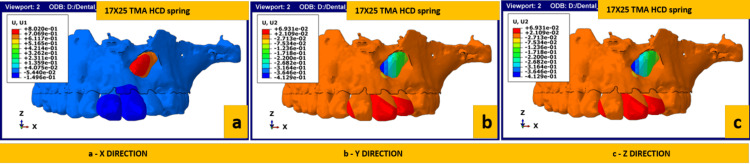
Rate of canine displacement using the HCD spring in (a) X-axis (sagittal), (b) Y-axis (transverse) and (c) Z-axis (vertical) TMA, titanium molybdenum alloy; HCD spring, Harsha's Canine Disimpaction spring

**Figure 6:**
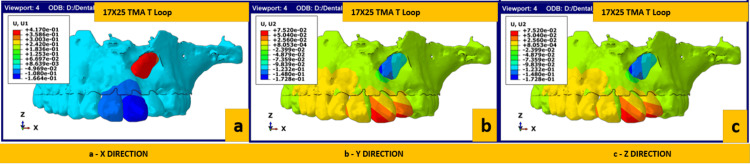
Rate of canine displacement using the T loop in (a) X-axis (sagittal), (b) Y-axis (transverse) and (c) Z-axis (vertical) TMA, titanium molybdenum alloy

Figures 5-6 depict the rate of displacement of the canine when activated with the HCD spring and T loop, respectively. The total number of activations required for complete disimpaction was calculated mathematically by considering the total amount of disimpaction needed (in mm) and the amount of disimpaction achieved with a single activation (in mm). Based on these calculations, the overall average activation required in the X-axis was 12-13 for the HCD spring and 25 for the T loop. The overall average activation in the Y-axis was 0.5 for the HCD spring and 1-1.5 for the T loop; the overall activation in the Z-axis was 89-90 for the HCD spring and 114 for the T loop. The overall activation required for disimpacting the canine requires about 12-13 activations on average using the HCD loop and 80 activations on average using a T Loop as per the mathematical calculation.

## Discussion

Canine impactions are treated using various protocols that include orthodontic disimpaction, extraction and auto-transplantation. The severity of the impaction and complexity of the treatment protocol requires precise diagnosis and treatment planning. Surgical exposure and disimpaction using orthodontic forces are the most commonly implemented protocol for the treatment of impacted canines [15]. Owing to the length of the root, the position of the canine in the oral cavity and the total surface volume, impacted canines pose varying levels of challenges during disimpaction. This can be overcome by using customized springs, or other force delivery systems. Loops or springs are commonly used methods for disimpaction of impacted teeth. They work under the principle of frictionless mechanics thereby providing greater anchorage control when compared to friction mechanics [16]. Though frictionless mechanics is technique sensitive, it brings about controlled tooth movement that is highly essential in cases of disimpaction as the tooth is closely related to adjacent tooth structures. Loops also give us the advantage of having a discriminate force system that helps us to closely monitor and quantify the extent and degree of tooth movement when compared to sliding mechanics.

Although there are some FEM studies that have been conducted with respect to canine disimpaction springs specifically for palaltal impactions, there are no reported studies, to our knowledge, regarding springs used for the disimpaction of bucally impacted canines. Hence, the present FEM study was done to evaluate the efficiency of the novel HCD spring utilised for the disimpaction of canine, compared to the T loop. The FEM method was chosen because of its advantages, such as simplicity of application, high speed, and repeatability [17,18].

The properties of the materials and textures were the most important factors that affected the stress distribution in FEM studies, and the modulus of elasticity of the springs, bone and Poisson's ratio were critical distinguishing features. The evaluated structures were assumed to be homogeneous, isotropic, and linearly elastic, and models were made based on this concept. In practical use, it is not possible for any 3D structure or material to be completely homogeneous and isotropic, unlike clinical conditions, which must be treated with caution. The outcomes in clinical conditions may differ depending on the age, bone thickness, quality and complexity of the malocclusion of the patient [19].

In the present study, two simulated models were formed, and the amount of movement of the impacted maxillary canine in three directions of space and stress values at adjacent teeth and bone were measured. The HCD spring brought about twice the amount of tooth movement when compared to the T loop. This could be attributed to the design of the spring. The increased length generated by incorporating two helices in the spring brought about three-dimensional movement of the impacted tooth on activation. This in turn provided a resultant force acting in all three planes providing significant tooth movement. When compared to previous studies by Murugesan and Ramasamy [20] and Chacko et al. [12], it was observed that springs with an increased length of wire incorporated in its design produced a greater moment-to-force ratio that enabled significant bodily movement. The HCD spring has an increased wire length incorporated into its structure with the help of coils and loops when compared to the T loop.

The distance between the two arms of the spring always plays an important role in predicting the amount of force generated on activation of the loop [21]. As the distance decreases and the two arms are closer to each other, the amount of force generated is reduced. This was attempted to be circumvented by the HCD spring by utilizing a longer span of wire between the two activation helices. The second feature was that the HCD spring was designed such that its components did not impinge the vestibular tissue and caused less trauma or damage to the underlying soft tissues. The same is not possible with the T loop as the design of the loop would not permit the same and hence greater tissue and vestibular trauma was expected [21].

There are few FEM studies related to impacted canines, but most of them are related to palatally impacted canines [17,22-24]. As per the results of these above-mentioned studies, it could be noticed that different inferences were obtained on the amount of stress experienced by adjacent structures and the rate of displacement of the tooth. This could be attributable to differences in the number of nodes, model designs, and forces used in FEM studies.

FEM analysis is a preferred modality to study the force and stress distribution during the eruption of impacted canines because of its advantages, such as application simplicity, quick results, and repeatability [23-25]. Though an FEM study can assess the force systems and stresses associated with canine disimpaction, it is still static and cannot replicate the dynamic and biological changes that take place in the surrounding structures during active tooth movement. These biological changes are not constant and are governed by various other systemic factors. This is one limitation of the present study. Hence, clinical evaluation should be carried out to further understand the role of confounding factors in influencing tooth movement. The moment-to-force ratio of each spring per activation should also be evaluated to further understand the biomechanics of tooth movement. Periodontal changes and also time intervals between activations required for disimpaction of the canine through the attached gingiva should also be evaluated clinically.

## Conclusions

This FEM study concluded that the HCD spring required 12-13 activations when compared to the T Loop, which required activation up to 80 times to bring about similar tooth movement. The HCD spring also brought about greater tooth movement compared to the T loop. Increased stress concentration in the disto-cervical region of the tooth and bone intersection region was observed with both HCD spring and T loop.
